# J. Young’s Early Maladaptive Schemas and Symptoms of Male Depression

**DOI:** 10.3390/life12020167

**Published:** 2022-01-24

**Authors:** Jan Chodkiewicz, Mateusz Wydrzyński, Monika Talarowska

**Affiliations:** Institute of Psychology, University of Lodz, 91-433 Lodz, Poland; jan.chodkiewicz@now.uni.lodz.pl (J.C.); mateusz.wydrzynski@edu.uni.lodz.pl (M.W.)

**Keywords:** male depression, early maladaptive schemas, GSDS-26

## Abstract

**Aim:** There are more non-specific, hence harder to diagnose, symptoms in the picture of male depression. These symptoms are strongly linked to social norms and roles traditionally assigned to men. The aim of this study was to assess the interrelationship of early maladaptive schemas that affect the formation of self-image as a man with indicators of male depression. **Materials and methods:** The Gender-Sensitive Depression Screening (GSDS-26) by A.M. Möller-Leimkühler and the Early Maladaptive Schema Questionnaire by J. Young (YSQ-S3-PL) were used. A group of 75 men (aged 18 to 50) were qualified to take part in the research. **Results:** The total score of the GSDS-26 scale and individual indicators of male depression are strongly positively correlated with the severity of all five domains of the YSQ-S3-PL questionnaire. The highest correlation coefficient value was obtained in the following areas: “Disconnection and rejection” (0.741), “Other-directedness” (0.711), and “Overvigilance and inhibition” (0.711). In case of the GSDS-26 total score and the following indicators—Elevated stress, Aggressiveness, Emotional control, Risky behavior, and Classic symptoms of depression—positive statistically significant associations were confirmed with each of the 18 schemas from the YSQ-S3-PL questionnaire. Multiple regression results revealed that the following domains were significant for symptoms typical of male depression: “Disconnection and rejection” and “Impaired autonomy and performance”. The “Impaired limits” area was found to be statistically significant only for symptoms of classic depression. **Conclusions:** (1) The GSDS-26 scale scores show positive associations with each domain of the YSQ-S3-PL questionnaire. (2) The following areas seem to be more important for atypical depressive symptoms in men: “Disconnection and rejection” and “Impaired autonomy and performance”, while for classic depression: “Impaired limits” was more important. (3) In therapeutic work with male depressive symptoms, it is useful to focus on dominant maladaptive schemas alongside beliefs about stereotypical male roles.

## 1. Introduction

It is widely recognized that depression is a heterogeneous entity. An individualized approach to understanding the etiology of depression seems to be increasingly important [1,2]. It is especially important to take into account gender differences in the etiology and clinical course of this disease [3]. However, for many decades, they focused primarily on the mental health of women. Attempts were made to explain differences in the prevalence of symptoms of anxiety disorders, eating disorders, specific personality disorders, or depressive disorders with gender dimorphism [4]. Issues related to the mental health of men received little attention. These differences have begun to gradually blur over the past 10 years [5]. It has been pointed out that the social, health, and economic costs of untreated mental disorders in men are much more severe than for women [6]. The risk of a successful suicide attempt (according to the National Police Headquarters in Poland, 85% of all deaths resulting from a suicide attempt in 2020 were male deaths [7]; similar values are observed in other European countries [8]) as well as the risk of developing an addiction to psychoactive substances is significantly higher among men [9,10].

The observations mentioned above have drawn the researchers’ attention to the issue of depression in men. It has been noted that there may be more non-specific symptoms in the picture of male depression that are less noticeable to the surroundings and therefore more difficult to diagnose. Furthermore, these symptoms are strongly associated with social norms and roles traditionally attributed to men (Table 1) [11].

Such a conceptualization of male depression led to attempts to study it and, consequently, to the development of appropriate diagnostic methods. The best known tool is the Gotland Male Depression Scale (GMDS) by Rutz [14]. Another scale, namely the Gender-Sensitive Depression Screening (GSDS-26) by A.M. Möller-Leimkühler, has been gradually gaining popularity. This scale—unlike the GMDS—allows for the assessment of various dimensions of male depression (they are described below in the section dedicated to the methodology of the presented study) [15].

The prevalence of depressive disorders and the social costs associated with them have prompted numerous studies on their etiology. In addition to biological and social factors, particular attention is given to the role of early childhood experiences such as emotional abuse, neglect, and unmet needs. Numerous meta-analyses have shown that these experiences are important predictors of depression occurring in both adolescence and adulthood e.g., [16,17].

The concept that seems to explain the mechanism and first of all the dynamics of disorder formation on the basis of early childhood experiences is the theory of early maladaptive schemas (EMS) created by Jeffrey E. Young [18]. The theory, which belongs to the so-called third wave of cognitive–behavioral therapy, emphasizes the importance of early childhood experiences in the formation of mental disorders as well as the need to refer in therapy to the history of development and life of the given patient [19].

According to the assumptions of EMS, the experiences we make in our earliest stages of development shape relatively stable patterns of functioning and beliefs about ourselves, other people, and the world around us. These patterns are referred to as schemas. In his theory, Young described 18 maladaptive schemas, grouped into five areas (domains) based on unmet needs. Failure to meet or inadequately meet one (or more) of the child’s basic developmental needs (so-called core needs) becomes the primary source of dysfunctional schemas [20]. Failure to meet these needs causes emotions that are difficult for the child, such as anxiety, anger, shame, or guilt. In an effort to avoid experiencing them, individuals engage in a variety of behavioral and coping strategies that—while reducing tension—also contribute to the perpetuation of the schemas [21]. 

Numerous studies confirm the associations of selected schemas with the onset of symptoms of recurrent depressive disorder, bipolar affective disorder, suicidal tendencies, obsessive–compulsive disorder, social phobia, addictions, and of course personality disorders and self-injury tendencies e.g., [22,23,24,25,26,27,28,29,30,31]. However, not enough studies focusing on early maladaptive schemas typical of male depression have been conducted so far [32].

Taking into account the aforementioned dependence, the aim of this study was to assess the interrelationships between J. Young’s early maladaptive schemas affecting the development of self-image as a man and indicators of male depression assessed using the GSDS-26 scale.

## 2. Method

Due to the current epidemiological situation, the research—whose results are presented herein—was conducted fully anonymously and online using a Google form, in the period between September and December 2020. The respondents (adults only) were enrolled by means of the ‘snowball’ method. The research procedure was conducted in accordance with the Declaration of Helsinki of the World Medical Association [33] and the ethical codes of the Belmont Report [34]. The study was approved by the Bioethical Committee of the Medical University of Lodz no: RNN/254/19/KE. 

Selected methods of descriptive statistics and methods of statistical reasoning were applied in the statistical analysis of the collected material. Spearman’s rank correlation coefficient and stepwise multiple regression coefficient were used to assess the relationship between the analyzed variables [35]. All statistical calculations were performed using STATISTICA PL computer software, version 13.3.

A self-reported questionnaire served to collect sociodemographic data. In addition, the following tools were applied:

1. Gender-Sensitive Depression Screening (GSDS-26) by A.M. Möller-Leimkühler. The scale consists of 26 statements allowing for the assessment of both typical symptoms of depression (one dimension) and symptoms that make up the five dimensions of male depression, i.e., increased levels of experienced stress, aggressiveness, emotional control, alcohol abuse, and engaging in risky behavior. The answers given are rated on a four-point scale (0–3). The Polish adaptation of the method characterized by satisfactory psychometric properties was applied [15,36].
2. J. Young’s Early Maladaptive Schema Questionnaire (YSQ-S3-PL) in the Polish adaptation by Oettingen et al. [37]. The method examines the intensity of each of the 18 schemas based on the self-report of a respondent, who is asked to respond to highlighted statements. This allows the pattern of schemas characteristic of a particular person to be identified. The questionnaire consists of 90 test items (5 for each schema). The scores for each schema are in the range of 5 to 30. The arithmetic mean for each schema and the total score for all are also calculated. When analyzing the results obtained in the YSQ-S3-PL questionnaire, a division into 5 schema areas selected by the author was used, i.e., Disconnection and rejection, Impaired autonomy and performance, Impaired limits, Other-directedness, Overvigilance and inhibition [38]. The Polish version of the method is characterized by acceptable psychometric properties [37].

## 3. Material

Study participants were enrolled by means of the ‘snowball’ method. Inclusion/exclusion criteria were as follows: age between 20 and 45 years, giving informed consent to participate in the study, no history of other Axis I or Axis II psychiatric disorders (other than a diagnosis of a depressive episode in the past).

A group of 75 men (aged 18 to 50) were qualified to take part in the research. The mean age of the respondents was 25.5 years (*SD* = 5.68). The characteristics of sociodemographic variables are presented in Table 2. Table 3 presents information regarding the past psychiatric treatment of the subjects. Those subjects who at the time of the examination or in the past declared a psychiatric diagnosis other than depressive episode and recurrent depressive disorder and were treated for those reasons were excluded from participation in the study.

Only 9 men in the study group are or were treated in the past for depressive disorder symptoms.

## 4. Results

The results recorded in the GSDS-26 scale and the YSQ-S3-PL questionnaire in the studied group are presented in Table 3.

In order to look at the associations of male depressive symptoms with early maladaptive schemas, a correlational analysis was conducted using the Spearman’s rho correlation coefficient. First, analyses were performed for the five domains of early maladaptive schemas (Table 4), which was followed by individual 18 schemas.

As Table 4 shows, the overall score of the GSDS-26 scale and individual indicators of male depression are strongly positively correlated with the severity of all five domains of the YSQ-S3-PL questionnaire. The highest correlation coefficient value was obtained for “Disconnection and rejection”, “Other-directedness”, and “Overvigilance and inhibition”. This means that failure to satisfy the need for security, empathy, stability, acceptance, and respect during childhood (“Disconnection and rejection”), the need to express one’s needs and emotions, which leads to excessive focus on others’ desires, emotions, and reactions at the expense of one’s own needs (“Other-directedness”), and the need for spontaneity (“Overvigilance and inhibition”) may be particularly relevant to the onset of male depressive symptoms.

With respect to the GSDS-26 subscales, the weakest correlations were recorded for the following two indicators: alcohol abuse and tendency for risky behaviors. Furthermore, the GSDS-26 scale indicator related to alcohol abuse significantly correlates only with the aforementioned area of “Other-directedness” and the area of “Impaired autonomy and performance”. This may be a consequence of the selection of the research group-people treated for reasons other than depressive disorders, including those with a diagnosis of personality disorders or substance abuse, were excluded.

A detailed analysis of the results of correlation of the 18 maladaptive schemas with the GSDS-26 scale scores confirmed the above analyses. In case of the GSDS-26 total score and the following indicators—Elevated stress, Aggressiveness, Emotional control, Risky behavior, and Classic symptoms of depression—positive statistically significant associations were confirmed with each of the 18 schemas from the YSQ-S3-PL questionnaire. In contrast, for the alcohol abuse indicator, a positive statistically significant but weak correlation was confirmed only for the “Emotional inhibition” schema (Rho = r = 0.241, *p* = 0.04). This schema manifests itself in the form of behaviors such as exaggerated inhibition of spontaneous actions, feelings, and communication with others in order to avoid disapproval from others, feeling of shame, or loss of control.

The next step in the statistical analyses was to assess the significance of the five areas of maladaptive schemas on the severity of the GSDS-26 scale indicators. For this purpose, the progressive stepwise multiple regression method was used to perform statistical calculations (Table 5).

The value of the “Elevated stress levels” indicator is associated in nearly 40% with one domain of early maladaptive schemas, namely “Other-directedness”. In contrast, the score volatility for the “Aggressiveness” indicator is explained in 43% by one schema area as well, namely “Impaired autonomy and performance”. 

“Disconnection and rejection”, “Impaired autonomy and performance”, and “Overvigilance and inhibition” play a significant role in case of the GSDS-26 scale indicator called “Emotional control”. Volatility of the “Emotional control” indicator is explained by these three domains in 45%.

The two indicators of the GSDS-26 scale only slightly explained by the values of the YSQ-S3-PL questionnaire are “Alcohol abuse” and “Risky behaviors”. As before, this phenomenon can be explained by the specifics of the selection of the study group (alcohol abusers and people with personality disorder diagnoses were excluded). However, it is noteworthy that the single significant predictor for both of these indicators was again found to be the “Impaired autonomy and performance” domain.

Another indicator, namely “Classic symptoms of depression”, is explained in nearly 50% by two domains of the YSQ-S3-PL questionnaire, i.e., “Disconnection and rejection” and “Impaired limits”. It is noteworthy that the “Impaired limits” domain only appears to be a predictor of classic symptoms of depression, not atypical ones.

The overall score of the GSDS-26 scale can be explained in more than 60% in the male subjects by the variability of two domains, i.e., “Disconnection and rejection” and “Other-directedness” (in this case, the result is at the level of central tendency).

Summarizing the results obtained, it is worth noting that among the five domains of early maladaptive schemas, the regression analysis most often includes “Impaired autonomy and performance” (four GSDS-26 scale indicators) and “Disconnection and rejection” (three GSDS-26 scale indicators). 

## 5. Discussion

As with personality traits, schemas are an indispensable part of a person’s mental structure. They have the nature of unconditional beliefs, not questioned by the given person; they constitute an important part of the person’s identity, his or her knowledge about themselves, other people, as well as about the surrounding world. Their strength, reinforcement, and frequency of activation determine the impact they have on the daily functioning of the given person [39]. However, their presence in itself does not indicate a disorder; everyone uses schemas. When faced with challenges that arise, different types of schemas can be activated to effectively deal with the difficult situation and the emotions that result. However, for maladaptive schemas formed in childhood, their activation in adulthood negatively affects how information is acquired, encoded, and stored, leading to dysfunctional emotions and behavioral responses [40]. Thus, the response to the activation of these schemas is usually part of their maintenance mechanism, which hinders the possibility of change [41,42]. 

The aforementioned observations are confirmed by numerous studies indicating that scores obtained in the Early Maladaptive Schema Questionnaire are a reliable and relatively stable marker of depressive disorders [43,44,45]. Approximately 60% of patients with depressive symptoms achieve a symptomatic improvement following the use of psychotherapy in the form of schema therapy [46,47]. 

According to Shorey et al. [48], early maladaptive schemas are stronger predictors of depression in men than in women. In the aforementioned study, the authors found statistically significant associations between the five schema domains and 11 schemas (out of 18) and the severity of depressive symptoms in men. Among the female subjects, only the “Other-directedness” area and two out of 18 non-adaptive schemas were found to be significant for the severity of depressive symptoms [48]. Male respondents also had significantly higher summed scores in each schema domain compared to females [48]. The classic symptoms of depression, regardless of gender, are in turn associated with the domains of “Disconnection and rejection”, “Impaired autonomy and performance”, and “Other-directedness” [49]. Thus, it would be worthwhile to test in the future how such a comparison between the sexes looks with the application of the method used in this paper to study depression.

In a longitudinal, 9-year study involving patients with a diagnosis of depressive disorder, Wang et al. [44] found a nearly 60% correlation between the areas of “Disconnection and rejection” and “Impaired limits” and the severity of depressive symptoms. It is noteworthy that in this study, the “Disconnection and rejection” domain was found to be a predictor of scores on two depression subscales and the GSDS-26 scale total score. In contrast, interestingly and worthy of further exploration, the domain of “Impaired limits” was found to be a predictor only of classic symptoms of depression and not of atypical ones. 

On the other hand, Tezel et al. [50] observed an association between an impaired communication style and the aforementioned domains of maladaptive schemas. The “Impaired limits” area is also associated with health risk behaviors [46]. According to Sedlinská et al. [51], patients with symptoms typical of male depression more often present personality traits from the so-called B cluster than from the A and C clusters, which also correlates with the aforementioned maladaptive schemas typical of men.

How can we link early maladaptive schemas to symptoms of male depression? The need to conform to traditional cultural and social norms are said to be some of the reasons for atypical depression in men. These norms for men relate to independence, bravery, competitiveness, mental and physical toughness, self-control, and the ability to suppress feelings and avoid anything “feminine” [52,53]. Some authors also claim that these norms include the need for risk taking, aggressiveness, multiple sexual encounters and conquests, dominance over women, explicit and manifested attachment to work, and gaining and maintaining social status [54,55]. Relationships may also include the ability to detect signs of depression in oneself (men who strictly adhere to the norm about the need to control emotions have more difficulty both recognizing their own lowered mood and consciously feeling grief and sadness) [1]. Moreover, people who believe that “problems should be dealt with on one’s own”, much later (if at all) ask for help from those around them, which may significantly worsen the personal, family, and financial situation of the patient, prolong the duration of therapy, and increase the risk of suicide. What is more, Sundag et al. [56] and Zeynel and Uzer [57] demonstrate that early maladaptive schemas are passed from generation to generation through parenting styles and coping strategies preferred by caregivers. Thus, cultural patterns and social norms associated with stereotypical male behaviors may be key in perpetuating maladaptive schemas leading to symptoms of male depression. However, confirmation of this relationship requires further research, including longitudinal studies.

The study presented herein demonstrated associations of typical and atypical depressive symptoms with different domains of maladaptive schemas according to Young. This means that the higher severity of maladaptive schemas included in them favors the occurrence of both types of depressive symptoms in men. The relationship between the intensity of maladaptive schemas and the severity of typical depression (most commonly measured by Beck’s BDI) has already been demonstrated for both men and women e.g., [45,58], and it is also supported by a recent large meta-analysis [40]. However, there has been no confirmation of these relationships for atypical depression to date.

Analyzing the obtained correlations, it is worth paying special attention to the “Impaired autonomy and performance” domain, which shows associations with such atypical and frequent symptoms of depression in men as aggressiveness, emotional control, alcohol abuse, and risky behavior. In an attempt to characterize people scoring high in this domain, we can see that they often lack self-confidence, fear failure in their achievements, and feel threatened and dependent. Such individuals were treated overprotectively as children, with the belief that they could not cope without loved ones, and their confidence and independence was not fostered [18,20]. Therefore, it may be hypothetically assumed that such a way of upbringing contributes in these men (especially in the situation of increased life demands and unavoidable confrontation with male role norms) to high levels of frustration and helplessness, suppression of emotions instead of searching for solutions, which may be alleviated with alcohol or “regulated” through emotional discharges and thus lead to the occurrence and deepening of depressive symptoms. Confirmation of this hypothesis obviously requires appropriate research. 

The mentioned relationship also leads to therapeutic conclusions. When working therapeutically with symptoms of male depression, it is useful to focus on beliefs about stereotypical male roles and behaviors as well as dominant schemas. This can both improve the therapeutic relationship (patients will be less likely to hide their symptoms and not be ashamed of them) and reduce the risk of depression recurrence [11]. Focusing on the schema domains identified in this study (“Disconnection and rejection” and “Impaired autonomy and performance”) may also have positive implications for the prevention of male depression [40]. In addition, the study highlights the point and importance of a careful analysis of dominant symptoms present in men experiencing depression, as different schemas and domains may be responsible for their occurrence, which may have serious implications for therapy effectiveness.

## 6. Limitations

Some of the limitations of the study include the relatively small study group and the participation of virtually only men without depressive symptoms. However, the mean overall score of the subjects obtained on the GSDS-26 scale is *M* = 18.8. The cut-off point for the German version of the scale is 18 points [15]. In the future, it is advisable to examine the associations of depressive symptoms with maladaptive schemas in men with (current and past) depressive disorders.

## 7. Conclusions

1. The GSDS-26 scale scores show positive associations with each domain of the YSQ-S3-PL questionnaire.
2. For atypical depressive symptoms in men, the following areas seem to be more important, namely “Disconnection and rejection” and “Impaired autonomy and performance”, while for classic depression, “Impaired limits” is more important.
3. In therapeutic work with male depressive symptoms, it is useful to focus on dominant maladaptive schemas alongside beliefs about stereotypical male roles.

## Figures and Tables

**Table 1:** Classic symptoms of depression and symptoms of male depression [12,13].

Classic Symptoms of Depression	Symptoms of Male Depression (So-Called Atypical Depression)
Sadness Being worried Insecurity Sense of helplessness and hopelessness	Outbursts of anger Abuse of psychoactive substances (alcohol, nicotine, drugs) Risky behavior Fatigue Feeling of tension combined with a decrease in resistance to stress Symptoms of professional burnout Avoidance of contact with others (including family relationships) Impulse control disorders (to a degree not previously present) Unspecified somatic symptoms

**Table 2:** Characteristics of the examined group—sociodemographic variables (*N* = 75).

	*N =* 75	%
Place of residence		
Rural settlement	12	15.58
Town with up to 100,000 inhabitants	17	22.57
City with more than 100,000 inhabitants	46	61.85
Education		
Primary	1	1.33
Vocational	2	2.67
Secondary	32	42.67
Higher	40	53.33
Marital status		
Bachelor	41	54.67
Married	25	33.33
Civil partnership	8	10.67
Legal separation	-	-
Divorced	1	1.33
Widower	-	-
Employment		
I study/learn	50	66.67
I don’t work	4	5.32
Permanent employment	21	28.1
Retirement/pension	-	-

**Table 3:** Severity of depressive symptoms and early maladaptive schemas in the study group (*N* = 75).

Variables	M	SD	Min.	Max.	Skewness	Kurtosis
GSDS-26						
Sum	18.81	10.92	0	41	0.181	−0.726
Elevated stress levels (5 items)	5.32	3.45	0	14	0.381	−0.373
Aggressiveness (6 items)	2.62	2.89	0	12	1.574	2.165
Emotional control (4 items)	5.34	3.28	0	12	0.113	−0.931
Alcohol abuse (3 items)	1.15	1.65	0	7	1.429	1.390
Risky behavior (3 items)	0.76	1.66	0	6	2.164	3.453
Classic symptoms of depression (5 items)	3.61	3.12	0	12	0.694	−0.286
YSQ-S3-PL						
Sum	243.71	75	90	425	−0.042	−0.518
disconnection and rejection						
Sum	66.47	25.81	25	145	0.331	−0.251
Emotional deprivation	11.81	5.86	5	30	0.661	−0.161
Abandonment/instability	13.93	6.21	5	28	0.366	−0.607
Mistrust/abuse	13.86	5.78	5	30	0.496	0.159
Defectiveness/shame	11.44	6.25	5	30	0.163	0.053
Social isolation/alienation	15.41	6.29	5	29	0.897	−0.838
impaired autonomy and performance						
Sum	44.47	18.22	20	87	0.471	−0.564
Dependence/incompetence	11.36	5.26	5	26	0.709	−0.101
Vulnerability to harm or illness	11.76	5.52	5	27	0.461	−0.666
Enmeshment/undeveloped self	10.23	4.91	5	23	0.748	−0.219
Failure to achieve	11.12	5.36	5	27	0.873	0.359
impaired limits						
Sum	29.49	8.13	10	50	0.011	0.224
Entitlement/grandiosity	15.28	4.81	5	29	0.331	0.391
Insufficient self-control/self-discipline	14.21	5.05	5	30	0.395	0.481
other-directedness						
Sum	43.44	14.02	15	69	−0.169	−0.711
Subjugation	11.73	5.62	5	27	0.648	−0.466
Self-sacrifice	15.49	5.58	5	28	0.123	−0.414
Approval seeking/recognition seeking	16.31	5.61	5	28	−0.208	−0.522
overvigilance and inhibition						
Sum	59.84	17.53	20	99	−0.163	−0.344
Negativity/pessimism	14.49	5.67	5	26	0.173	−0.816
Emotional inhibition	15.09	5.92	5	29	−0.067	−0.777
Unrelenting standards/hypercriticalness	16.96	5.26	5	29	0.134	−0.151
Punitiveness	13.29	5.44	5	28	0.217	−0.468

GSDS—Gender-Sensitive Depression Screening; YSQ-S3-PL—Young Schema Questionnaire; M—mean; SD—standard deviation; Min.—minimum value; Max.—maximum value.

**Table 4:** Values of Spearman’s rho correlation coefficient for the GSDS-26 scale scores and five domains of the YSQ-S3-PL questionnaire.

Variables							
	GSDS-26						
	Elevated Stress Levels	Aggressiveness	Emotional Control	Alcohol Abuse	Risky Behavior	Classic Symptoms of Depression	Sum
YSQ-S3-PL Sum	0.595 *	0.573 *	0.508 *	0.197	0.382 *	0.726 *	0.775 *
YSQ-S3-PL/Area							
Disconnection and rejection	0.511 *	0.547 *	0.566 *	0.161	0.325 *	0.693 *	0.741 *
Impaired autonomy and performance	0.575 *	0.579 *	0.361 *	0.237 **	0.354 *	0.648 *	0.696 *
Impaired limits	0.473 *	0.405 *	0.427 *	0.182	0.341 *	0.673 *	0.655 *
Other-directedness	0.625 *	0.518 *	0.394 *	0.236 **	0.374 *	0.641 *	0.711 *
Overvigilance and inhibition	0.556 *	0.489 *	0.503 *	0.131	0.342 *	0.665 *	0.711 *

GSDS—Gender-Sensitive Depression Screening; YSQ-S3-PL—Young Schema Questionnaire; *-*p* ≤ 0.001; **-*p* ≤ 0.05.

**Table 5:** Progressive stepwise regression coefficient results for GSDS-26 scale scores (dependent variables) and five domains of the YSQ-S3-PL questionnaire (independent variables).

Variable	GSDS		
	Elevated Stress Levels		
	R^2^	b	*p*
Intercept term	0.396	−1.977	0.086
Other-directedness		0.106	0.005 *
	Aggressiveness		
	R^2^	b	*p*
Intercept term	0.432	−2.299	0.002 *
Impaired autonomy and performance		0.075	0.002 *
	Emotional Control		
	R^2^	b	*p*
Intercept term	0.447	−0.191	0.854
Disconnection and rejection		0.097	0.001 *
Impaired autonomy and performance		−0.098	0.001 *
Overvigilance and inhibition		0.057	0.048 *
	Alcohol Abuse		
	R^2^	b	*p*
Intercept term	0.086	−0.039	0.936
Impaired autonomy and performance		0.027	0.011 *
	Risky Behavior		
	R^2^	b	*p*
Intercept term	0.154	−0.834	0.081
Impaired autonomy and performance		0.035	0.001 *
	Classic Symptoms of Depression		
	R^2^	b	*p*
Intercept term	0.489	−3.558	0.001 *
Disconnection and rejection		0.053	0.001 *
Impaired limits		0.122	0.007 *
	GSDS-26 Sum		
	R^2^	B	*p*
Intercept term	0.633	−9.016	0.002 *
Disconnection and rejection		0.161	0.003 *
Other-directedness		0.182	0.061

GSDS—Gender-Sensitive Depression Screening; YSQ-S3-PL—Young Schema Questionnaire; *-*p* statistically significant.

## Data Availability

Institute of Psychology, University of Lodz, Lodz, Poland.
